# Territorial and gender-linked risk factors for Buruli ulcer in Southern Benin: A case-control study using geographic and behavioral surveying

**DOI:** 10.1371/journal.pntd.0013509

**Published:** 2025-09-08

**Authors:** Harvey Johnson, Alexandra Boccarossa, Esai Anagonou, Télésphore Brou, Perin Catraye, Sébastien Fleuret, Estelle Marion, Matthieu Eveillard

**Affiliations:** 1 INCIT, Inserm, Univ Angers, CHU Angers, Angers, France; 2 ESO, CNRS, Univ Angers, Angers, France; 3 University Abomey Calavi, Cifred, Benin; 4 Geography Department, University of La Reunion, Saint-Denis, France; 5 CDTLUB Raoul Follereau, Pobe, Benin; Institut Pasteur, FRANCE

## Abstract

**Objectives:**

The manuscript examines the risk factors associated with Buruli ulcer in endemic regions of Benin, focusing on community practices, agricultural activities, and age and gender disparities.

**Methods:**

The study, conducted from November 2021 to June 2024, used a prospective case-control approach combined with a geographic health survey. The study involved home interviews followed by guided tours of areas frequented by participants, allowing the precise identification of practices at risk of Buruli ulcer. Univariate analysis and stepwise backward stepwise logistic regression were carried out.

**Results:**

Overall, 117 patients and 234 controls were included. Multivariate analysis showed that activities such as bathing (OR = 3.2, p = .04), farming in flooded areas (OR = 3.8, p = .02), and frequenting irrigation canals (OR = 5.0, p = .003) were independent risk factors for Buruli ulcer. The originality of this study was that these risks were not distributed in the same way across territory and depended on age and gender.

**Conclusion:**

The findings suggest that public health interventions need to consider gender, age, territorial features, and local agricultural practices. Furthermore, integrating geographical and spatial data into epidemiological studies offers valuable insights helping to prevent the spread of this neglected tropical disease.

## Author summary

Buruli ulcer is a skin disease caused by a bacterium that lives in aquatic environments. It mostly affects specific rural areas in West and Central Africa, usually near rivers surrounded by large, flooded, and cultivated plains. People likely get infected during activities involving water, such as swimming, bathing, collecting unprotected water, or farming in flood-prone zones. Our study, conducted in southeastern Benin, involved interviewing patients and healthy individuals about their daily habits. We also walked with them along the paths they regularly use. This helped us identify different risk factors. For example, recreational swimming is mainly a risk factor for young boys. Other risks depend on how the land is used—for instance, the presence of irrigation canals. These findings show that prevention strategies should be tailored to each area and adapted to different groups of people based on age and gender.

## Introduction

Buruli ulcer (BU) is a neglected tropical disease and is the third most common mycobacterial infection after tuberculosis and leprosy. *Mycobacterium ulcerans*, a mycobacterium of environmental origin, is responsible for BU [1]. This infectious disease is a necrotizing subcutaneous disease causing large ulcerations of the skin and soft tissues, as well as disabilities if diagnosed at advanced stages of the disease [1].

Most endemic areas are in West and Central Africa and southeastern Australia [2]. The mechanisms of *M. ulcerans* transmission remain not fully understood, although several hypotheses have already been suggested. In Africa, the infection is acquired from a *M. ulcerans*-contaminated environmental aquatic source [3], with data suggesting inoculation into the skin through sources such as water bug bite or some other puncture [4–6]. Concurrently, in Australia, an animal reservoir (possums) has been identified, and Buruli ulcer is considered a zoonosis, with mosquitoes playing the role of a mechanical vector for inoculation [7,8]. In contrast, to date, no aquatic-associated mammal has been identified as a potential reservoir of the bacterium.

In West Africa, several epidemiological studies have highlighted the existence of a strong association between frequenting wet environments and *M. ulcerans* acquisition. Living near aquatic ecosystems and engaging in water-related activities have already been reported as risk factors for acquiring the disease [9–14]. Similarly, daily attendance at natural water points for domestic activities was identified as a risk factor for acquiring the disease, while using protected water from water drilling for these activities was a protective factor [15,16]. It has also been demonstrated that the risk is higher when people encounter slow-flowing streams or stagnant water points via case-control or landscape studies [13,15,17–19].

In Benin, the incidence of new cases began to decrease in 2010, confirming a trend observed on a global scale in Africa [16,20]. In 2019, we showed that regular use of well water for domestic activities was protective against Buruli ulcer [16]. We then identified several types of human behavior associated with a higher risk of transmission through contact with unprotected water sources [15]. Our previous specific and refined results provided a broader scope for the design of an appropriate preventive strategy, including certain practices or infrastructures observed during our field investigations. However, this strategy could be improved by the addition of knowledge about irrigation practices and agricultural work in low-lying areas. Furthermore, another epidemiological characteristic in these areas is that male children are more exposed to the disease, while adult women are more infected than adult men [15,21]. These data suggest the potential existence of territorial practices potentially associated with age and gender, which could put individuals more at risk of BU.

In this context, our main objective was to identify living spaces and community practices, including agricultural practices, susceptible to increasing the risk of BU acquisition in this endemic area. Particular attention will be paid to the distribution of age, gender, and territories in the various activities described. To achieve this goal, we have implemented a new method consisting of a prospective case-control study combined with a geographical survey.

## Methods

### Ethics statement

Due to the low literacy rate in the population affected by the disease, an information notes and informed consent to participate in the study were read orally by the investigator or a translator proficient in the different languages spoken (Goun, Fon, Holi, Nago) in southeastern Benin. Then, formal consent was signed by all participants or by the legal representative for minor participants (parent/guardian). Participants were free to withdraw from the study at any time. The study was submitted to an ethics committee at the Ministry of Health in Benin and approved under the reference: 0583/CLERB-UP/P/SP/R/SA.

### Study design

The study took place over nearly three years (from November 2021 to June 2024). The methodology used in this study was based on a method called the “geographic health survey,” specifically developed for BU field investigations [22]. The survey included a home interview (lasting between 45 minutes and 1 hour) with questions related to the respondent’s personal characteristics and lifestyle habits, including mobility patterns. Then, all activities involving contact with water and frequented places were examined in the field by the investigators during guided tours planned over half a day, during which they commented on-site about the practices and attitudes related to the places mentioned in the interview.

### Recruitment of cases and controls

**Fig 1:**
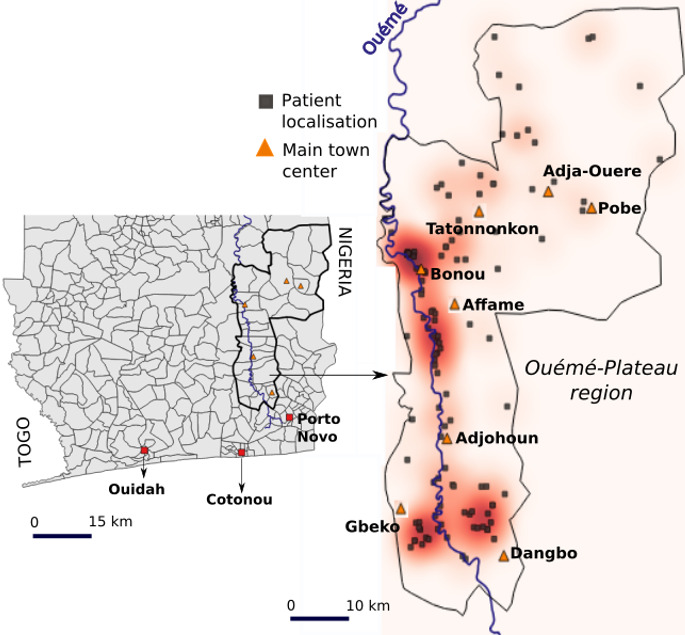
Study area encompassing the departments of Ouémé and Plateau in Benin, with black squares indicating the locations of patients’ residences. Base layer provided by Ministry of Territory Planning of Benin (https://gadm.org/download_country_v3.html).

Cases were individuals treated for Buruli ulcer at the Center for diagnosis and treatment of leprosy and Buruli ulcer (CDTLUB) in Pobè, which is located at the border with Nigeria, after clinical examination and confirmation by qPCR according to the WHO criteria [23]. To be recruited into the study, cases had to reside in one of the five municipalities in the study area, which included the two departments of Ouémé and Plateau in southeastern Benin (Fig 1). We chose to exclude children under eight years from the cohort. Indeed, in the preparatory phase of the study, we observed that young children had more difficulty recalling retrospective memory or staying focused during the entire survey phase (an entire morning or afternoon). For children aged 8–15, the use of fun data-gathering aids was employed (drawing cards). For each case, two controls were recruited after matching for age (± 2 years), gender, and place of residence (preferably the same neighborhood). Controls must never have contracted Buruli ulcer in their lifetime, nor belong to the same family as the cases. The random walking procedure used here was adapted from the methods detailed in a previous WHO survey [24], which are easy to implement during field investigations in rural areas. We visited the nearest house along the random trajectory and listed all members of the household, to identify potential controls fulfilling the matching criteria. If several suitable individuals were identified, we selected the person most closely matching the criteria for the case concerned. The procedure was repeated until two suitable controls were identified for each case.

### Questionnaire structure

Locally, the population speaks either Fon or Yoruba, with varying degrees of proficiency in French. In this context, an interpreter with a perfect command of the 2 local languages systematically accompanied the interviewer. The questionnaire contained four main parts: 1) identity and life story, 2) representations and stories of the disease, 3) individual habits and living spaces, and 4) mobilities, i.e., living and working areas and travel. The questionnaire was designed to assess lifestyle habits during the past year. The individual habits section was divided into three topics: domestic activities and living spaces, activities outside the home, and details about farming activities. The section on farming practices was developed in this study. It considers the specific features of the areas studied. If the land was in flood-prone areas, a specific question was asked as to whether the respondent continued to farm when the water level was high. The respondent was also asked whether they practiced agroforestry, for example, combining the palm grove with market gardening. In each part, there were both closed and open questions. During the guided visits, the answers to certain questions could be clarified and detailed, most often in the mobilities section.

### Statistical analyses

For the qualitative data, the interview at the respondent’s home was recorded and transcribed using SONAL software (CetuEthic univ-Tours), enabling certain points in the discourse to be coded a posteriori. The data were processed and analyzed with Epi-Info version 7.2.5.0 (Centers for Disease Control, Atlanta, USA) and IBM Statistical Package for Social Sciences (SPSS) version 25. Proportions were expressed for all study variables. Odds ratios, 95% confidence intervals for these odds ratios, and p-values were calculated during the univariate analysis. A p-value < 0.05 was considered significant. For multivariate analysis, a conditional logistic regression model was used. Variables with a p-value < 0.20 were introduced into this model to simultaneously examine their independent effect.

### Spatial analysis

Patients’ homes were collected manually in the field using a Garmin eTrex 22x GPS unit. The heat map was created using the QGIS “heat map” extension, which can graphically represent and match the intensity of patient grouping to a color intensity at the scale of the study area (Fig 1). Altitude data were collected from Landsat’s 30-meter SRTM (Shuttle Radar Topography Mission). A functional land-use typology was produced, based on 7 distinct classes created specifically for the study: farmed fields, palm plantations, wooded vegetation, bare soils, built areas, permanent water, and wetland vegetation (Fig 5). The images were collected with a camera and a drone (Figs 4 and 5). All the maps in this study were produced with the Inkscape and QGIS software suites.

**Fig 2:**
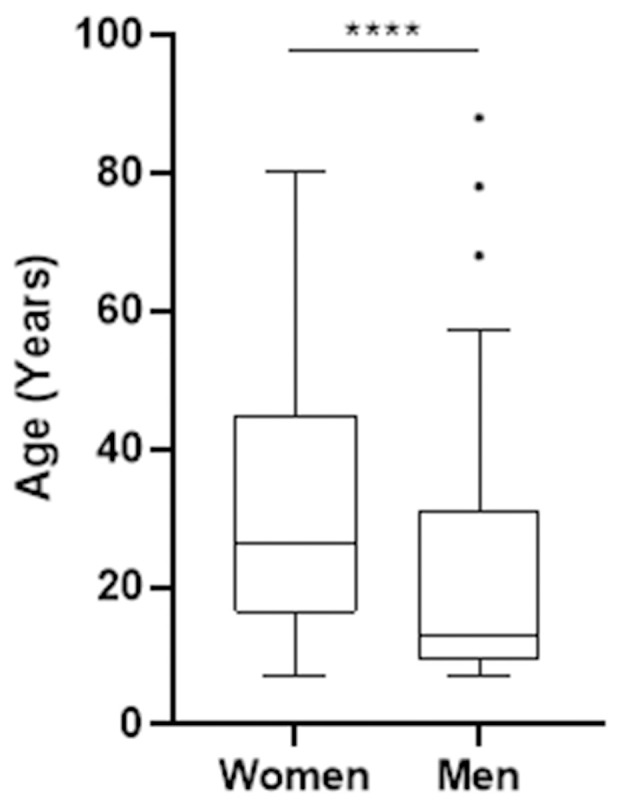
Age and gender distribution of the recruited cohort.

**Fig 3:**
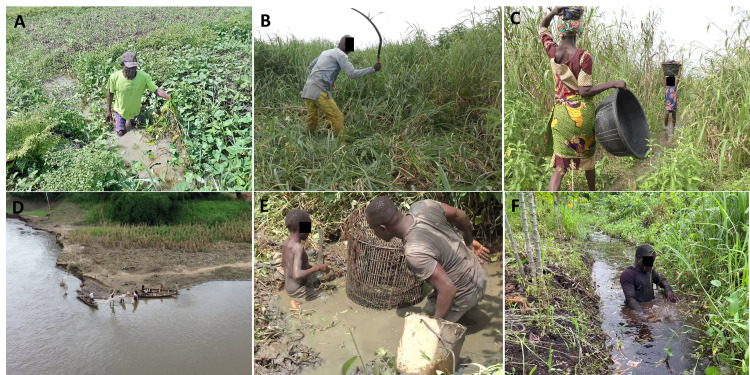
Photographs illustrating close contact with stagnant water during various activities. **A)** Manual collection of semi-aquatic plants in damp lowland zones for animal feed; **B)** Manual weeding with a machete, carried out by a man wearing long clothing; **C)** Transporting crops from the fields, a task predominantly performed by women and children; **D)** Fishing activity using a pirogue on the river; **E)** Fishing using traps in swamps; **F)** Fishing with nets in irrigation canals.

**Fig 4:**
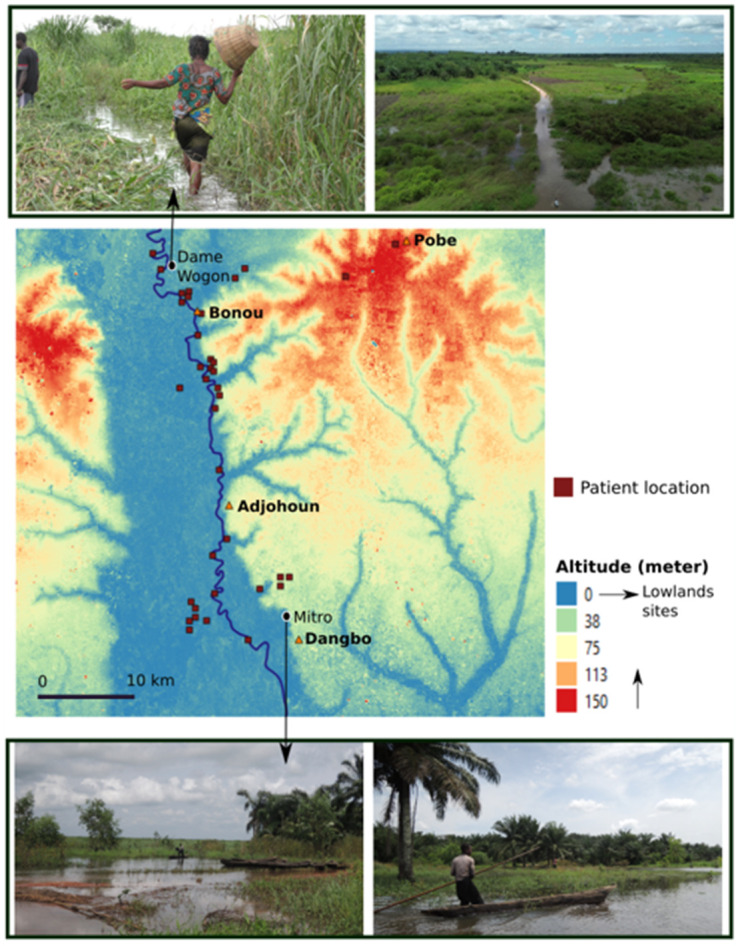
Territorial differences in weeding and harvesting activities during flood periods: These activities are more commonly practiced in the northern part of the study area due to topographical features. Datas for the altitude and land surface are available from https://www.naturalearthdata.com/.

**Fig 5:**
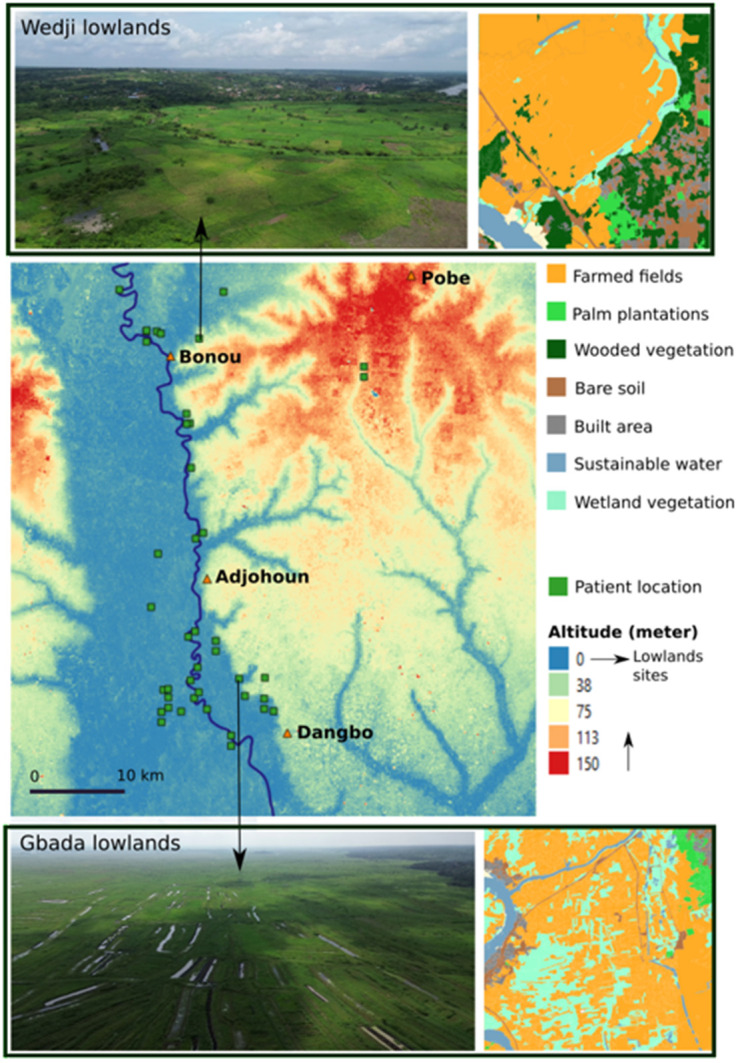
Territorial specificity of irrigation canal usage: This activity is more prevalent in the southern part of the study area. Spatial analysis shows a higher concentration of irrigation canals in the southern region compared to the northern region. Datas for the altitude and land surface are available from https://www.naturalearthdata.com/.

## Results

### Demographic characteristics of study participants

A total of 351 people participated in the geographic health survey, including 117 patients and 234 controls (Table 1). The majority of them (77.8%) lived along the Ouémé River, located in the department of the same name (Fig 1). Spatial representation of patients’ homes shows a distribution around 4 hotspots: 2 in the north of Ouémé around Bonou and Affame, and 2 in the south of Ouémé, around Gbeko and Dangbo (Fig 1 and Table 1). The male/female sex ratio was 0.72. Although the number of cases was generally higher among females, men were more frequently infected during childhood. There was a distortion in the age distribution by gender of the patients (Fig 2). Indeed, the median age of cases was 13 years for males and 27 years for females. 50.3% of males were under 13 years old, while this proportion among females was 14.2% (p < 0.0001).

**Table 1:** Socio-demographic features of the people included in the study.

Variables studied	Cases n = 117 (%)	Controls n = 234 (%)
**Age**		
≤13	35 (29,9)	68 (29,1)
>13	82 (70,1)	166 (70,9)
**Gender**		
Male	49 (41,9)	98 (41,9)
Female	68 (58,1)	136 (58,1)
**Residential department**		
Ouémé	91 (77,8)	182 (77,8)
Plateau	26 (22,2)	52 (22,2)
**Residential municipality**		
Adja-Ouèrè	18 (15,4)	36 (15,4)
Adjohoun	22 (18,8)	46 (19,7)
Bonou	45 (38,5)	88 (37,6)
Dangbo	24 (20,5)	48 (20,5)
Pobè	8 (6,8)	16 (6,8)

### Contribution of guided tours to the data collection

With the guided tour method, we observed some activities or behaviors that were not spontaneously declared or observed before. For example, manually collecting semi-aquatic plants in damp lowland zones to feed animals is an activity discovered during this study. The plants collected, known as water spinach (*Ipomoea aquatica* Forssk), are generally harvested without protective clothing (such as boots or long clothes), resulting in close or direct contact with the water and associated aquatic organisms, thus representing a potential risk practice (Fig 3A). In addition, this method allowed us to extend discussions outside the strict protocol of the interview. It enabled us to explore the territories and several practices in detail and identify additional or differential risk factors. For example, some farming activities are affected by the hazards of flooding. When the water rose, farmers harvested the last crops before the plots became impassable, without much gender distinction identified. While weeding and mowing grass with a scythe or machete were carried out by men using protective devices (Fig 3B), transporting crops from the fields to the dwellings was usually assigned to women, often accompanied by children. This transport often implied crossing flooded paths, was carried out several times daily, and usually without protective devices (Fig 3C). Finally, the guided tour method enabled us to realize that the same activity can be practiced in many ways and contact with the water can be either minimal or very significant, especially with fishing. The first fishing technique observed on the river involved little contact with the water, with the use of nets from the river or from a pirogue and was carried out mainly by men (Fig 3D). The second techniques observed required direct and prolonged contact with all or part of the body, for example, by immersing oneself completely to collect fish caught in nets placed in irrigation canals (Fig 3E, 3F). This level of detail is important and can only be achieved through multiple observations in the field and open discussions with local people.

### Associations between activities and acquisition of Buruli ulcer according to univariate analysis

#### Direct contact with water.

The associations between the activities of the surveyed individuals and Buruli ulcer disease are presented in Table 2. First, it appears that bathing, an activity primarily observed among children, is significantly associated with the disease (p = 0.0001), with the proportion of cases who engage in this activity twice as high as that of controls. In the questionnaire, bathing refers to a recreational activity. Individuals engaging in this activity are most often partially or fully unclothed. Another direct contact with water, crossing a flooded path to go to the field, seemed less associated with the disease, with a lower odds ratio and a difference at the limit of significance (p = 0.08).

**Table 2:** Association between participants’ activities and Buruli ulcer – univariate analysis.

Activities	% of cases	% of controls	OR (CI95%)	*P*-values
**Bathing**	30.8%	14.5%	2.6 (1.5 – 4.5)	0.0001
**Crossing a flooded path to get to the field**	55.0%	44.2%	1.5 (0.95 – 2.5)	0.08
**Agroforestry practice**				
On dry land	59.3%	54.9%	1.2 (0.6 – 2.3)	0.59
On flooded land	40.9%	19.5%	2.9 (1.6 – 5.1)	0.0001
**Specific agricultural activities**				
Field clearing	44.3%	52.2%	0.73 (0.45 – 1.2)	0.20
Sowing	93.1%	93.8%	0.89 (0.34 – 2.3)	0.81
Weeding and harvesting on flooded lands	63.4%	46.2%	2.0 (1.1 – 3.5)	0.02
Transporting the crops	95.1%	95.3%	0.97 (0.32 – 2.9)	0.95
Collecting palm nuts	58.9%	47.0%	1.6 (0.99 – 2.7)	0.06
Grass collection for animals	41.3%	15.0%	4.0 (2.2 – 7.3)	0.0001
Processing the harvest production in the field	49.6%	54.7%	0.81 (0.52-1.26)	0.36
**Other activities in the field**				
Partial or total immersion in water points	68.6%	57.0%	1.7 (1.0 – 2.7)	0.04
Partial or total immersion in an irrigation channel	57.7%	35.2%	2.5 (1.4 – 4.6)	0.002
**Other activities**				
Fishing/ aquaculture	27.4%	32.1%	0.83 (0.51-1.36)	0.46
Sand mining	8.5%	11.1%	0.75 (0.35-1.61)	0.46
Industrial and craft works	51.3%	57.7%	0.77 (0.49-1.21)	0.26

**CI95%, confidence interval 95%.**

#### Farming activities.

Participants practiced both agroforestry and palm grove activities (Table 2); we were interested in the association between this practice of different types of crops and the disease, differentiating the activities carried out on dry land from those carried out on wetlands. No association exists when the different types of crops are carried out on dry land (p = 0.59). On the contrary, the proportion of cases with wetland crops was more than twice as high as compared to controls (40.9% vs. 19.5%; p = 0.0001). At the level of agricultural tasks, weeding and collecting grass for animals were significantly associated with the disease (p = 0.02 and p = 0.0001, respectively), with an odds ratio of 4.0 for the latter activity (Fig 3A). The association between collecting palm nuts and disease was at the limit of significance (p = 0.06). On the other hand, clearing, sowing, and transporting crops were not associated with the disease (Fig 3B and 3C).

#### Others.

Participants in the study could have other water-related activities during their field activities, such as frequenting water points, irrigation canals, or more specifically fishing in irrigation canals (Fig 3F). These three types of activities were significantly associated with the disease (Table 2).

### Associations between activities and acquisition of Buruli ulcer according to multivariate analysis independent risk factors

The results of the multivariate analysis are presented in Table 3. The variables significantly and independently associated with the disease were: bathing (OR = 3.8, p = 0.032), weeding and harvesting on flooded land (OR = 5, p = 0.012), frequenting an irrigation canal in the field (OR = 4.7, p = 0.007), and practicing agroforestry on wetland (OR = 3.3, p = 0.04). We can note that all adjusted odds ratios were greater than 3, showing strong associations.

**Table 3:** Association between participants’ activities and Buruli ulcer – multivariate analysis.

Activities	Adjusted OR (CI95%)	*P*-values
Bathing	3.8 (1.1 – 12.9)	0.032
Weeding and harvesting on flooded lands	5.0 (1.4 – 17.6)	0.012
Partial or total immersion in an irrigation channel in the field	4.7 (1.5 – 14.3)	0.007
Agroforestry practice on flooded lands	3.3 (1.0 – 10.5)	0.043
Grass collection for animals	1.2 (0.85-1.59)	0.276
Collecting palm nuts	1.277 (0.44-3.72)	0.65
Partial or total immersion in water points	1.377 (0.693-2.734)	0.361
Crossing a flooded path to get to the field	1.07 (0.560-2.042)	0.838

### Comparison of independent risk factors across territories, age groups, and gender

We then wanted to know whether the identified risk behaviors were carried out across the entire study area, regardless of age or gender. The four risk factors identified by multivariate analysis were differently distributed among BU cases according to the limit we used for defining the north and south of the study area and according to age and gender (Figs 1 and 2). All stratified analyses are presented in a table for additional information (S1 Table).

Bathing activity was as common in the north and south of the study area (29.6% vs. 30.4%). It was identified as a risk factor in women of all ages, whereas it was not commonly practiced by them. Concurrently, bathing was a common practice among men. While it was identified as a risk factor in boys under 13, it was not significantly associated with BU cases in men above 13 (Table 4).

**Table 4:** Associations between practicing mixed crops on flooded land, bathing activity, and BU cases according to age and gender.

Variables and stratifications	Number of participants (cases + controls)	OR	*P*-values
***Mixed crops on flooded land***			
Overall	68		
Men			
Overall	30	3.5	<0.01
< 13	23	4.0	0.01
≥ 13	7	3.1	0.20
Women			
Overall	38	2.4	0.02
< 13	8	1.1	1
≥ 13	30	3.1	< 0.01
***Bathing***			
Overall	123		
Men			
Overall	103	2.1	0.07
< 13	40	4.7	< 0.01
≥ 13	63	1.5	0.53
Women			
Overall	20	3.4	< 0.01
< 13	8	7.0	0.02
≥ 13	12	3.0	0.06

Agroforestry on wetlands was more often practiced in the north than in the south of the study area, but the difference was not significant (48.1% vs. 29.4%, respectively; p = 0.08). This activity was mainly a risk factor in men of all ages and in women above 13 (Table 4).

Continuing to go to the fields during flood periods was more often recorded in the BU cases living in the northern part of the area (71.7% vs. 48.0%; p = 0.04), and particularly among women (Table 5). We have observed that people in the south use pirogues much more frequently when flooding occurs, whereas in the north, due to the topography, pirogues are only present at the river level and not in flooded fields (Fig 4).

**Table 5:** Associations between frequenting irrigation channels, continuing to go to the fields during the flood periods and BU cases, according to the gender and the localization in the north or in the south of the study area.

Variables and stratifications	Number of participants (cases + controls)	OR	*P*-values
***Frequenting irrigation channels***			
Overall	193		
Men			
Overall	90	2.8	0.02
< 13	47	2.9	0.09
≥ 13	43	2.6	0.15
Women			
Overall	103	2.3	0.04
< 13	15	0.5	0.7
≥ 13	88	2.9	0.02
North			
Overall	102	4.5	< 0.01
Men	51	3.6	0.07
Women	51	5.2	0.01
South			
Overall	91	3.0	0.03
Men	39	7.3	0.04
Women	52	2.0	0.26
***Continuing to go to the fields during flood periods***			
Overall	221		
Men			
Overall	103	1.2	0.72
< 13	59	1.1	0.93
≥ 13	44	1.3	0.68
Women			
Overall	118	3.4	< 0.01
< 13	20	6.0	0.12
≥ 13	98	3.1	0.01
North			
Overall	135	2.6	0.01
Men	70	1.4	0.51
Women	65	6.6	< 0.01
South			
Overall	86	1.2	0.65
Men	33	0.73	0.68
Women	53	1.75	0.36

Lastly, entering the irrigation channels was significantly and strongly more often observed in the BU cases living in the southern part of the area (80.6% vs. 40.0%; p < 0.001). Spatial analysis shows a much greater presence of irrigation canals in the south than in the north (Fig 5). This practice was significantly associated with BU among men of all ages (p = 0.02), especially in the southern part of our study area, and among women above 13 in the northern part of the area (p = 0.02) (Table 5).

## Discussion

This study aimed to identify living spaces and community practices that increase the risk of BU acquisition in endemic areas of Benin, with particular attention to gender and age disparities. The findings offer crucial insights into the potential risk factors in these communities and highlight several significant and independent associations between specific activities and BU acquisition.

One of the key findings of our study was the strong association between activities involving direct contact with water and the acquisition of BU. This aligns with previous studies in other endemic areas that have suggested the importance of water-related activities as a primary route for *Mycobacterium ulcerans* transmission [3,11,12,17]. Bathing, in particular, was identified as a significant risk factor for BU, especially among children as already described in *M. ulcerans* PCR-positive cohorts [25,26]. The observation that boys were particularly exposed to this risk during childhood is noteworthy, suggesting that gender-specific practices may play a role in *M. ulcerans* transmission. This gender heterogeneity in infected children could be linked to the different ways in which children of different genders engage with water sources or recreational activities. Moreover, it underscores the necessity for targeted interventions that consider age and gender differences in BU prevention strategies.

Fieldwork in flood-prone areas had already been identified as a risk factor in PCR-positive cohorts in Ghana and Cote d’Ivoire [27,28]. Another important finding of our study was the association between farming activities in flooded areas and BU, particularly among women. In the northern part of our study area, the topographical features seem more conducive to full or partial immersion in stagnant water bodies or slow-flowing streams while harvesting crops. This contrasts with the southern area, where people often use pirogues for transport during floods (Fig 4). The risk of BU acquisition was potentially compounded by the lack of protective measures, such as wearing boots or long clothing, during these activities, as observed during the guided tours. These findings demonstrate how environmental features, gender, and agricultural practices intersect to influence the risk of BU.

Agroforestry practices in wetlands were also significantly associated with BU acquisition, particularly among men and women over the age of 13. These findings reinforce the hypothesis that agricultural activities in flood-prone areas should be a key focus of preventive measures. Emphasizing the use of protective clothing and improving infrastructure to reduce exposure are essential strategies for mitigating these risks.

The frequenting of irrigation channels also emerged as a significant risk factor for BU acquisition, with geographical differentiation observed between the northern and southern parts of the study area. In the southern part, where irrigation channels are more widespread and cover larger portions of the land (Fig 5), individuals were more likely to engage in this activity (including fishing). This practice was strongly associated with BU among men in the south and women over the age of 13 in the north, pointing to territorial variations in disease risk that may be influenced by local environmental factors or community practices. The geographical distribution of these risk factors emphasizes the need for localized interventions that consider specific community practices, environmental conditions, and demographic characteristics.

One of the key strengths of our study methodology was the use of guided tours and spatial analysis. The guided tour method proved particularly valuable in identifying additional, unreported risk factors. For example, activities such as the collection of aquatic plants for animal feed and farming activities in flooded areas were not spontaneously reported in the survey but were observed to expose individuals to significant BU risks. These findings underscore the importance of integrating detailed geographical and environmental contexts into studies of pathogen transmission [18,19].

The creation of heat maps allowed for a clearer visualization of areas with higher concentrations of BU cases, helping to pinpoint high-risk zones that might benefit from intensified public health efforts. By coupling spatial data with epidemiological analysis, we gained a deeper understanding of the environmental and social dynamics driving pathogen transmission and were able to identify micro-configurations of high-risk BU acquisition. This approach is a valuable tool for public health authorities, enabling them to tailor interventions to the specific needs of at-risk communities.

This study has several limitations. First, it was conducted within a geographically limited area of Benin, in a specific local context, which restricts the generalizability of the findings. Applying the questionnaire in other endemic regions across Africa would help assess the broader relevance of the results. Second, the case-control design may be subject to residual confounding, as we cannot completely exclude the existence of other systematic differences between cases and controls beyond disease status. The frequency of activities was also not considered in our analysis. Nevertheless, such bias is unlikely given the recruitment procedures used. Finally, our findings support the hypothesis that Buruli ulcer acquisition may be influenced by gender- and age-specific water-related practices, shaped by local topography. A targeted action-research approach in well-defined areas would be valuable to test and refine these hypotheses.

In conclusion, our study underscores the complexity of *M. ulcerans* transmission, which is influenced by a combination of environmental, behavioral, and socio-demographic factors that vary by territory. The findings suggest that preventive interventions should not only focus on water-related practices in general, but also be adapted to the specific micro-configurations of risk resulting from the interplay of gender, age, and local agricultural and geographical features. Consequently, educational programs should be designed with this diversity in mind, offering targeted preventive measures. Additionally, improving access to safe water sources will play a critical role in reducing the incidence of BU. Finally, the integration of geographical and spatial data into epidemiological studies presents a promising approach for identifying and mitigating environmental risks, which could help prevent potentially other tropical diseases with significant environmental reservoirs.

## Supporting information

S1 Fig Flowchart of patient inclusion. (TIF)

S1 Table Results of stratified analysis of Tables 4 and 5.
(XLSX)
