# Supplementary figures and images for: Territorial and gender-linked risk factors for Buruli ulcer in Southern Benin: A case-control study using geographic and behavioral surveying
Source: PLoS Negl Trop Dis. 2025 Sep 8;19(9):e0013509. doi: 10.1371/journal.pntd.0013509 (PMC12431644; doi:10.1371/journal.pntd.0013509)

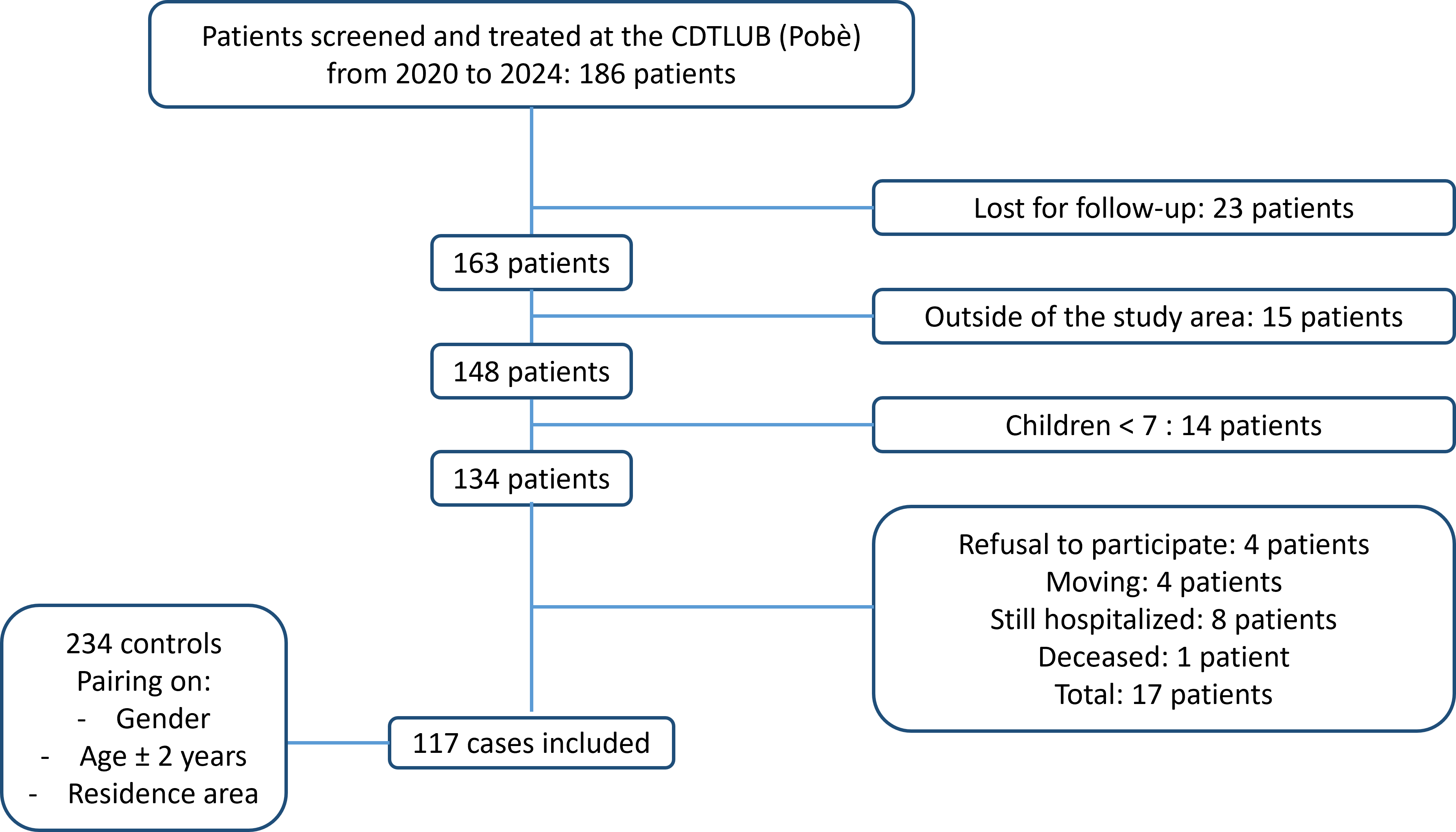

Supplement: S1 Fig — (TIF) [file pntd.0013509.s001.tif]
